# SARS-CoV-2 seroprevalence and antibody trajectories after easing of COVID-19 restrictions: a longitudinal study in China

**DOI:** 10.3389/fpubh.2024.1420993

**Published:** 2024-12-03

**Authors:** Feng Ling, Zenghao Xu, Jimin Sun, Xiaoxiao Wang, Yan Feng, Ying Liu, Yijuan Chen, Jinna Wang, Zhiping Chen, Kun Chen

**Affiliations:** ^1^Department of Public Health, Second Affiliated Hospital, Zhejiang University School of Medicine, Hangzhou, China; ^2^Zhejiang Provincial Center for Disease Control and Prevention, Hangzhou, China

**Keywords:** COVID-19, SARS-CoV-2, seroprevalence, trajectory, risk factor, China

## Abstract

**Background:**

We aimed to evaluate the seroprevalence of SARS-CoV-2 and investigate the trajectories of protective immunity and associated risk factors in eastern China between March and November 2023 after the easing of COVID-19 restrictions.

**Materials and methods:**

We conducted repeated population-based seroepidemiologic studies using a multistage, population-stratified, cluster random sampling method. We measured neutralizing antibodies (nAbs) using a fluorescence immunoassay. We calculated both overall and stratified seroprevalence. The latent class growth mixed model (LCGMM) was used to analyze the dynamic trajectories of antibodies, and a multinomial logistic regression model was used to identify factors associated with different antibody trajectory patterns.

**Results:**

A total of 6,147 participants were included at baseline, with a median age of 53.61 years. Both observed and adjusted seroprevalence remained high and stable throughout the study period. The LCGMM identified four distinct antibody trajectories: 75.22% of participants had a high and stable antibody trajectory, while nearly 8% of them exhibited an increase, decline, or low-stable antibody trajectory. Younger participants, women, those fully vaccinated, and individuals with a history of previous infection were more likely to have high and stable antibody trajectories.

**Conclusion:**

The majority of the population maintained sustained protective immunity after the outbreak, following the easing of COVID-19 restrictions across the country.

## Introduction

COVID-19 (1), caused by the severe acute respiratory syndrome coronavirus 2 (SARS-CoV-2) (2), has led to an unprecedented global pandemic crisis over the past 4 years. To date, there have been over 771 million confirmed cases and 6 million deaths worldwide. Asymptomatic infections account for up to 40% of cases (3), with silent transmission playing a significant role in outbreaks (4–7). While reverse transcription-polymerase chain reaction (RT-PCR) and rapid antigen assays have facilitated the diagnosis of both symptomatic and asymptomatic infections (8), serologic testing remains crucial for assessing the presence and longevity of antibodies after infection or vaccination.

Studies of antibody dynamics have shown varying results, with some reporting a decline in neutralizing antibodies (nAbs) over time after infection or vaccination (9–11), while others observed sustained high levels, particularly in vaccinated individuals (12, 13). Research using latent class growth mixture models (LCGMM) has classified antibody response trajectories as distinct patterns, offering insights into how different populations maintain immunity over time (14). However, the majority of these studies were conducted during the early stages of the pandemic with relatively small sample sizes. Limited data are available after the easing of COVID-19 restrictions, which is important for designing future immunization strategies. On 7 December 2022, China eased its stringent COVID-19 control measures, leading to the rapid emergence of new outbreaks, predominantly of the SARS-CoV-2 Omicron lineages BA.5.2 and BG.7 across the country (15). Despite high vaccination rates, there is limited knowledge regarding the long-term persistence of immunity in the population after the easing of restrictions.

To address these knowledge gaps, we conducted a prospective, longitudinal seroepidemiologic study to investigate the seroprevalence of SARS-CoV-2 and explore the trajectories of nAbs after the easing of COVID-19 restrictions in China.

## Materials and methods

### Study design and participants

**Figure 1:**
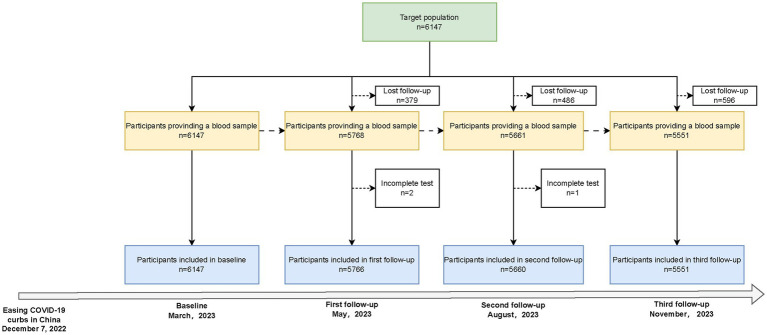
Flowchart of the study population.

This repeated population-based seroepidemiologic study was conducted between March 2023 and November 2023 in Zhejiang Province, China, which is administratively divided into 11 cities, 90 counties (districts), and over 20,000 villages (communities). We employed a multistage, population-stratified, cluster random sampling method with the following steps: First, in each of the 11 cities in Zhejiang Province, China, 1 or 2 counties (districts) were randomly selected. Second, in each county (district) of 11 cities, 2 villages (communities) were randomly selected, and a total of 24 sampling sites were identified (Supplementary Figure S1). Finally, age-specific random sampling was conducted in each village (community), sample size calculation was based on a cross-sectional design, and 500 residents were included in each county (Supplementary methods). The baseline survey was conducted in March 2023, with three follow-ups in May, August, and November 2023. The flowchart for the inclusion and exclusion of study participants is shown in Figure 1. Written informed consent was obtained from all participants, with an impartial witness facilitating the process for those who were unable to read and write. The study was approved by the Ethics Committee of the Zhejiang Provincial Center for Disease Control and Prevention (2023-005-01).

### Epidemiology data collection

At baseline, face-to-face questionnaires were used to collect participants’ demographic information (age, sex, and ethnicity), comorbidities, and vaccination history. Information on SARS-CoV-2 infection history, including time of onset and severity, was also collected. Vaccination data were retrieved from the National Vaccine Registry.

### Laboratory procedures

Venous blood samples were collected from each participant and transported to the local Center for Disease Control and Prevention (CDC) laboratory. After centrifugation, the serum was aliquoted under aseptic conditions in a biosafety cabinet and stored at −20°C. The samples were then transported under a cold chain to the Microbiology Laboratory, Zhejiang Provincial Center for Disease Control and Prevention, Hangzhou, China.

Neutralizing antibodies (nAbs) were detected using the COVID-19 nAbs FIA kit from Assure Tech. (Hangzhou) Co., Ltd. This kit, a fluorescence immunoassay, directly detects nAbs against the wild strain of SARS-CoV-2 in human whole blood, serum, or plasma specimens. The membrane of the test strip is pre-coated with the human ACE2 receptor protein on the test region, while the SARS-CoV-2 RBD antigen conjugated to fluorescent particles is on the conjugate pad. During testing, 80 μL of the serum sample was added to the strip’s sample well and incubated in a 37°C incubator for 15 min. Following incubation, the test strip was inserted into an AFI-6000 Fluorescence Immunoassay Analyzer (Assure Tech. (Hangzhou) Co., Ltd.), with the result displayed in terms of inhibition rate (IR). In accordance with the manufacturer’s instructions, an IR of ≥20% was considered seropositive for SARS-CoV-2 nAbs, while an IR of <20% was considered seronegative. According to the clinical evaluation of the characteristics, the kit demonstrated a sensitivity of 0.96 and a specificity of 1.00, as validated against virus neutralization experiments.

### Quality control

Epidemiology data were recorded in a unified data platform, with quality control overseen by experienced public health physicians. For the serologic test, given the shift in the predominant SARS-CoV-2 strain to the XBB strain of the Omicron lineages post-easing restrictions, we randomly selected 918 samples from three follow-ups to assess the IR of nAbs against the XBB strain. Spearman’s rank correlation was performed to analyze the relationship between the IR of nAbs against the wild strain and the IR of nAbs against the XBB strain, with the results illustrated in Supplementary Figure S2. A significantly positive correlation (*p* < 0.001) was observed, suggesting that the nAbs against the wild strain could serve as an indicator of the nAbs level against the XBB strain.

### Statistical analysis

Continuous variables were described using a median with an interquartile range (IQR), whereas categorical variables were presented as frequencies and percentages. Seroprevalence was calculated as the proportion of individuals with a positive result relative to those with completed tests at baseline and three follow-ups, and the 95% confidence interval (CI) was estimated based on a binominal distribution. While adjusting for test kit performance, the adjusted seroprevalence was calculated using the following formula:


$$\mathrm{Adjusted}\;\mathrm{prevalence}=\frac{\mathrm{Observed}\;\mathrm{Prevalence}+\mathrm{Specificity}-1}{\mathrm{Sensitivity}+\mathrm{Specificity}-1}$$

The bootstrap method was used to estimate the 95% CI of the adjusted seroprevalence.

A generalized additive model (GAM) was used to explore the non-linear relationship between age and seroprevalence. This model was selected over simpler linear models because it allows for greater flexibility when modeling complex, non-linear relationships between variables.

To assess the dynamics of nAbs, the latent class growth mixture model (LCGMM) was used to identify various longitudinal trajectories, which assumed that the population was heterogeneous and individuals in the study may follow different antibody response patterns (16). This approach allowed us to identify latent subgroups with varying antibody trajectories (e.g., persistently high levels, waning, or increasing over time) rather than assuming a uniform trajectory for all participants. In terms of model selection, different shapes, including linear, quadratic, and cubic, were tested to allow for flexibility in the longitudinal nAbs trajectories. We explored models with 1 to 5 classes and selected the best-fitting model based on the following criteria: (i) the lowest Bayesian information criterion; (ii) high mean posterior class membership probabilities (> 0.8); (iii) high mean posterior probabilities (> 0.7); (iv) a sufficient number of patients in each latent class (> 1%). We used multinomial logistic regression to explore the factors associated with different nAbs trajectories, reporting relative risk ratios (RRs) and 95% CIs.

All statistical analyses were performed in R software (version 4.1.3), with a statistical significance level set at a two-sided *p*-value of <0.05.

## Results

### Characteristics of the study population

Of the 6,147 participants who completed the baseline survey, 5,766, 5,660, and 5,551 subjects participated in the three follow-ups, with compliance rates of 93.80, 92.08, and 90.30%, respectively (Figure 1). The median age of the study population [IQR] was 53.61 [27.65] years at baseline and was similar across all follow-ups. Women comprised 54.35% of the participants, and more than 99% were of Han ethnicity. Urban residents were more than rural residents, and 67.95% reported no comorbidities, while 30.78% reported 1–2 comorbidities. COVID-19 vaccine coverage was 95.35%, with 76.61% fully vaccinated (≥ three doses) at baseline. During the follow-up, the vaccinated population did not substantially change. The self-reported SARS-CoV-2 infection rose from 75.26 to 78.15% during the study period (Table 1).

**Table 1:** Characteristics of the study population.

	Baseline (*n* = 6,147)	First follow-up (*n* = 5,766)	Second follow-up (*n* = 5,660)	Third follow-up (*n* = 5,551)
Age, years				
Median, [IQR]	53.61 [27.65]	53.42 [27.75]	53.78 [26.84]	53.90 [26.88]
<3	37 (0.60)	27 (0.47)	24 (0.42)	25 (0.45)
3–17	655 (10.66)	624 (10.82)	546 (9.65)	550 (9.91)
18–59	3,358 (54.63)	3,163 (54.86)	3,159 (55.81)	3,074 (55.38)
≥60	2097 (34.11)	1952 (33.85)	1931 (34.12)	1902 (34.26)
Sex				
Woman	3,341 (54.35)	3,116 (54.04)	3,067 (54.19)	3,032 (54.62)
Man	2,806 (45.65)	2,650 (45.96)	2,593 (45.81)	2,519 (45.38)
Ethnicity				
Han	6,130 (99.72)	5,751 (99.74)	5,647 (99.77)	5,536 (99.73)
Other	17 (0.28)	15 (0.26)	13 (0.23)	15 (0.27)
Region of residence				
Urban	4,630 (75.32)	4,332 (75.13)	4,252 (75.12)	4,171 (75.14)
Rural	1,517 (24.68)	1,434 (24.87)	1,408 (24.88)	1,380 (24.86)
Comorbidities				
0	4,177 (67.95)	3,922 (68.02)	3,830 (67.67)	3,749 (67.54)
1–2	1892 (30.78)	1770 (30.70)	1757 (31.04)	1728 (31.13)
≥3	78 (1.27)	74 (1.28)	73 (1.29)	74 (1.33)
Vaccinated against COVID-19				
Unvaccinated	286 (4.65)	253 (4.39)	244 (4.31)	246 (4.43)
One dose	101 (1.64)	95 (1.65)	93 (1.64)	93 (1.68)
Two doses	1,051 (17.10)	993 (17.22)	910 (16.08)	903 (16.27)
≥3 doses	4,709 (76.61)	4,425 (76.74)	4,413 (77.97)	4,309 (77.63)
Infection with SARS-CoV-2				
No	1,521 (24.74)	1,386 (24.04)	1,250 (22.08)	1,213 (21.85)
Yes	4,626 (75.26)	4,380 (75.96)	4,410 (77.92)	4,338 (78.15)
IQR: interquartile range.				

### Overall and stratified seroprevalence

The observed overall seroprevalence (95% CI) was 89.77% (88.98–90.51%) at baseline, increasing to 90.16% (89.37–90.92%), 90.80% (90.01–91.54%), and 90.47% (89.67–91.23%) at three follow-ups. The seroprevalence of the unvaccinated population was 45.10% at baseline, showing fluctuations at three follow-ups (27.27, 50.00, and 50.41%). Vaccinated individuals consistently displayed higher seroprevalence, ranging from 91.95 to 93.05%. The seroprevalence was further adjusted for test kit performance. Overall, the adjusted seroprevalence (95% CI) was 93.51% (92.89–94.11%) at baseline and 93.92% (93.30–94.50%), 94.58% (93.98–95.16%), and 94.24% (93.60–94.79%) at three follow-ups, with a slightly increasing trend. In the unvaccinated population, the adjusted seroprevalence (95% CI) was also significantly lower than in the vaccinated population. The age-weighted adjusted seroprevalence also showed similar results (Table 2).

**Table 2:** Overall and vaccination-stratified seroprevalence of SARS-CoV-2.

	Baseline (*n* = 6,147)	First follow-up (*n* = 5,766)	Second follow-up (*n* = 5,660)	Third follow-up (*n* = 5,551)
Observed seroprevalence, % (95% CI)				
Overall	89.77 (88.98–90.51)	90.16 (89.37–90.92)	90.80 (90.01–91.54)	90.47 (89.67–91.23)
Vaccinated against COVID-19				
Unvaccinated	45.10 (39.24–51.07)	27.27 (21.88–33.20)	50.00 (43.55–56.45)	50.41 (43.98–56.82)
Vaccinated	91.95 (91.22–92.63)	93.05 (92.35–93.71)	92.63 (91.91–93.32)	92.33 (91.58–93.03)
Fully vaccinated (≥ three doses)	92.89 (92.11–93.60)	94.51 (93.80–95.16)	93.48 (92.71–94.19)	93.13 (92.34–93.87)
Adjusted seroprevalence, % (95% CI)^a^				
Overall	93.51 (92.89–94.11)	93.92 (93.30–94.50)	94.58 (93.98–95.16)	94.24 (93.60–94.79)
Vaccinated against COVID-19				
Unvaccinated	46.98 (41.25–52.80)	28.41 (23.32–33.99)	52.08 (45.90–58.21)	52.51 (46.75–58.54)
Vaccinated	95.78 (95.24–96.25)	96.93 (96.46–97.39)	96.49 (95.99–96.97)	96.17 (95.65–96.68)
Fully vaccinated (≥ three doses)	96.76 (96.24–97.24)	98.45 (98.06–98.80)	97.37 (96.85–97.83)	97.01 (96.50–97.52)
Age-weighted adjusted seroprevalence, % (95% CI)^b^				
Overall	93.92 (93.91–93.94)	94.33 (94.31–94.34)	95.12 (95.11–95.13)	94.51 (94.50–94.52)
Vaccinated against COVID-19				
Unvaccinated	48.31 (48.28–48.34)	28.38 (28.35–28.40)	52.83 (52.80–52.86)	53.02 (52.99–53.04)
Vaccinated	96.54 (96.53–96.55)	97.64 (97.64–97.65)	97.28 (97.27–97.29)	96.75 (96.74–96.75)
Fully vaccinated (≥ three doses)	93.09 (93.07–93.10)	95.06 (95.05–95.08)	99.66 (99.66–99.66)	99.27 (99.26–99.27)

CI, confidence interval.

a Adjusted seroprevalence was calculated using the following formula: $\mathrm{Adjusted}\;\mathrm{prevalence}=\frac{\mathrm{Observed}\;\mathrm{Prevalence}+\mathrm{Specificity}-1}{\mathrm{Sensitivity}+\mathrm{Specificity}-1}$ , and the 95% CI was estimated by using the bootstrap method.

b Age weighting was based on the 7th National Population Census in 2020.

The results of the analysis of the relationship between age and seroprevalence are shown in Supplementary Figure S3. Overall, a significant non-linear relationship with age was demonstrated for seroprevalence (all *P*_for trend_ < 0.05), but not significantly higher in women compared to men. The fitted curve suggested an increase until approximately 50 years of age and a decline thereafter.

### Trajectories of nAbs and associated risk factors

**Figure 2:**
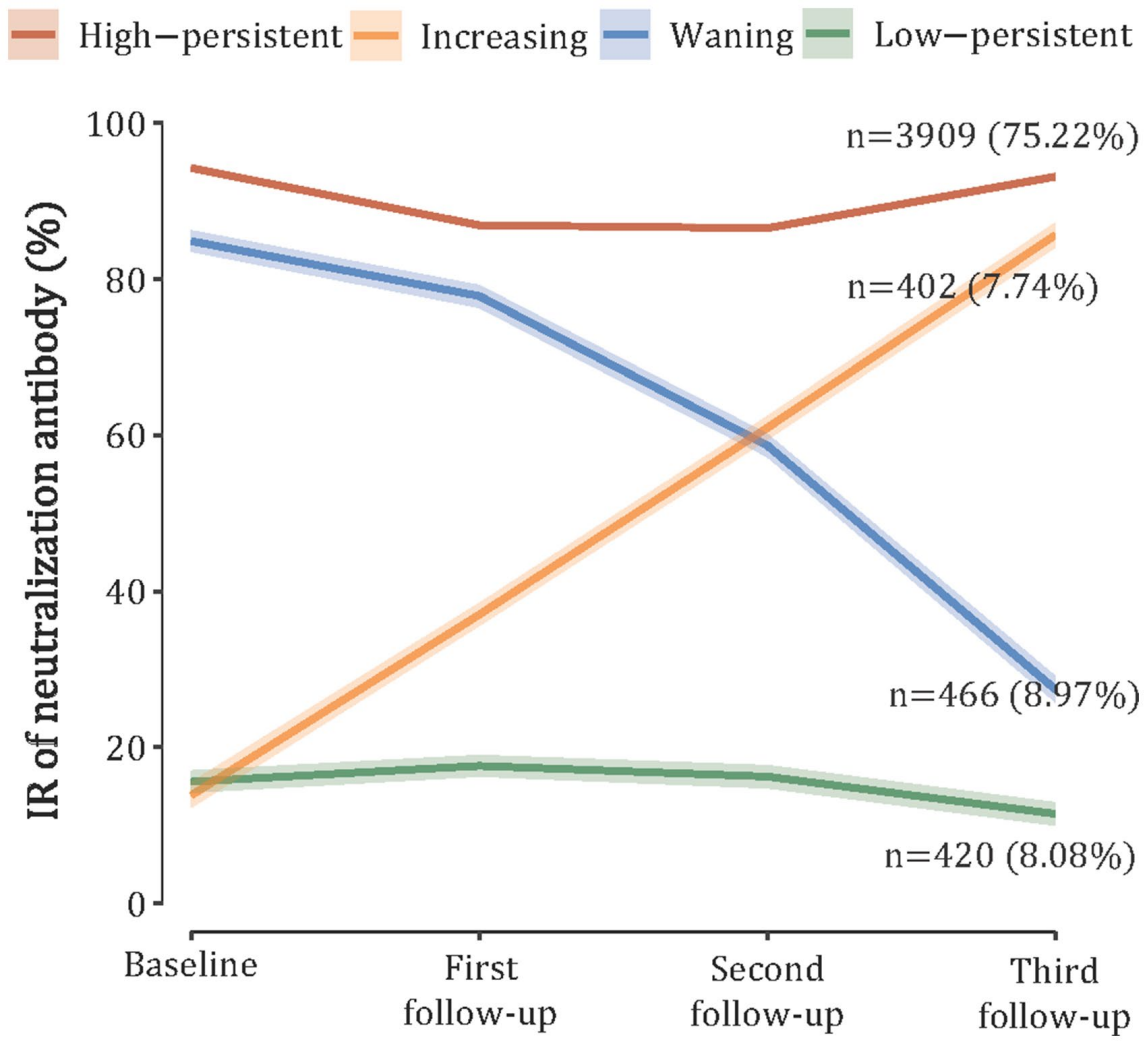
SARS-CoV-2 neutralizing antibody trajectories based on the latent class growth mixture model.

In the trajectory analyses, we included 5,197 (84.55%) participants with four serologic tests and identified four discrete trajectories of nAbs (Figure 2). Furthermore, 75.95% (*n* = 3,909) of participants maintained a high level throughout the study period (referred to as “high-persistent”), 7.81% (*n* = 402) of participants started with a low level and experienced a sustained increase (referred to as “increasing”), 9.05% (*n* = 466) of participants started with a high level and then declined (referred to as “waning”), and 8.16% (*n* = 420) of participants maintained a low nAbs level (referred to as “low-persistent”). Class membership probabilities were high (all >90%), suggesting that participants’ trajectories could be reliably assigned to one of the four classes (Supplementary Table S2). After stratification by age, we found that the adults (≥18 years) had a similar trajectory to the overall population, but the children and adolescents had a different trajectory (Supplementary Figure S4).

Characteristics of 5,197 participants classified by trajectory of antibody levels are presented in Supplementary Table S3. Participants with a “high-persistent” trajectory were more likely to be 18–59 years old, women living in urban areas, without comorbidity, fully vaccinated (≥3 doses), and having a previous history of SARS-CoV-2 infection.

**Figure 3:**
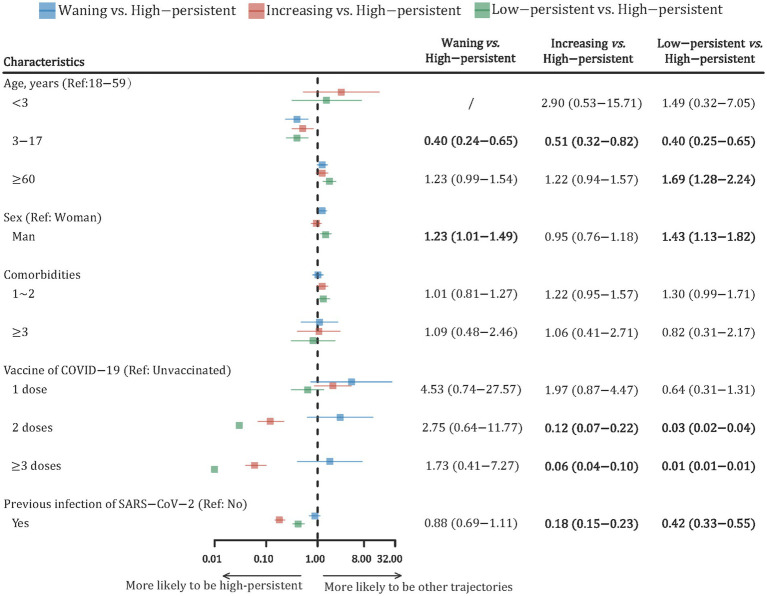
Forest plot of risk factors with the dynamic trajectories of SARS-CoV-2 neutralizing antibody based on the multinomial logistic regression model. The risk ratio (RR) and its 95% confidence interval (CI) were adjusted by all variables in the figure. RR, risk ratio; CI, confidence interval.

Furthermore, the multinomial logistic regression model was conducted to explore the association between potential risk factors and the trajectory of nAbs levels (Figure 3). Compared with participants aged 18–59 years, those aged 3–17 years were more likely to be in a “high-persistent” trajectory. Male participants had a 23% higher risk of being in a “waning” trajectory (RR: 1.23; 95% CI: 1.01–1.49) and a 43% higher risk of being in a “low-persistent” trajectory (RR: 1.43; 95% CI: 1.13–1.82) compared to female participants. There was no statistically significant difference in nAbs trajectories between participants with different numbers of comorbidities. Compared with participants who were not vaccinated, those who received two or more doses of the COVID-19 vaccine were more likely to have a “high-persistent” trajectory. Participants with previous SARS-CoV-2 infection were also more likely to be in the “high-persistent” class, with RRs (95% CIs) at 0.18 (0.15–0.23) and 0.42 (0.33–0.55), respectively.

## Discussion

In this longitudinal study, we found that SARS-CoV-2 seroprevalence remained consistently high, with more than 93% of the population exhibiting positive neutralizing antibodies (nAbs) throughout the study period. The vast majority of participants (75.95%) maintained a high-persistent antibody trajectory, while smaller groups showed increasing, waning, or low-persistent nAbs levels. Full vaccination, younger age, female sex, and a history of prior infection were associated with more favorable antibody trajectories (i.e., high-persistent). These findings highlight the sustained protective immunity in a highly vaccinated population following the easing of COVID-19 restrictions in China.

Our results have important implications for public health strategies, particularly concerning booster vaccination programs and monitoring of immune status in the population. The identification of risk factors for declining or low-persistent antibody trajectories (e.g., older age, male sex, and fewer vaccine doses) suggests that targeted interventions may be necessary to enhance long-term immunity in vulnerable subgroups. Seroprevalence of SARS-CoV-2 exhibits considerable variation across studies, which may be influenced by various factors, including geographic location, demographics, study timelines, testing methods, and test accuracy. Compared to a multicenter seroepidemiologic study in China that reported 89% seroprevalence after large-scale SARS-CoV-2 infection (*n* = 100), our findings indicated a comparable yet higher seroprevalence (17). Additionally, Zhou et al. reported seropositivity of more than 90% among children aged 8 months to 12 years in February and March 2023 (*n* = 1,065) (18). Consistent with prior research, our study emphasized the rapid spread of the COVID-19 epidemic in mainland China after the suspension of zero-COVID control measures (19). Studies conducted in other countries also confirmed a high seroprevalence of SARS-CoV-2 (>90%) after the COVID-19 epidemic. For instance, Sulcebe et al. conducted two consecutive cross-sectional studies and observed a rise in seropositivity from 70.9 to 92.1% in August 2021 and 2022 in Albania (20). Notably, we identified a significantly higher seroprevalence of SARS-CoV-2 in the vaccinated population compared to the unvaccinated population at baseline and three follow-ups. Despite the majority of participants having received the last dose of COVID-19 vaccine by the end of 2022, the high vaccination rate, with 95.35% of the study population receiving at least one dose and 76.61% fully vaccinated, also contributed to the high seroprevalence observed in our study.

nAbs exhibit a rapid rise following infection and can persist for years to decades due to long-lived plasma and memory B cells in most acute viral infections, playing important roles in viral clearance and defense against viral diseases (21). Previous studies on the dynamics of SARS-CoV-2 antibody levels mainly focused on the individual level or fitted with a linear regression model (13, 22–25). For instance, Kaygusuz et al. demonstrated that both IgG and nAbs levels continued unabated even after 9 months of follow-up (24), with no reinfections reported during the study period. Three studies have used a latent class mixed model to analyze antibody responses after vaccination or infection (14, 26, 27). Wei et al. included 7,256 COVID-19 patients in the United Kingdom between April 2020 and June 2021 and found that 64.5% of participants had a classical seroconversion trajectory, and 24.0% were seronegative non-responders (14). However, after the easing of COVID-19 restrictions and the resumption of social activities, the likelihood of recurrent infection appears inevitable. Thus, understanding the trajectories of antibodies in real-world settings has significant public health value because it can inform subsequent risks of outbreaks. Our study used repeated measurements and LCGMM to fit the trajectories of nAbs and provided valuable insights into within-person antibody responses after the easing of COVID-19 restrictions in mainland China. Notably, we observed that 75.22% of participants displayed a “High-persistent” trajectory of nAbs, while nearly 8% of participants had “increasing,” “waning,” and “Low-persistent” trajectories, respectively. Although no specific threshold of nAbs determined an individual’s risk of COVID-19 infection, prior studies have suggested that higher antibody quantities are associated with a decreased risk of subsequent symptomatic COVID-19 infection (28). The persistently high nAbs may be due to the asymptomatic infection since very few populations were vaccinated during the follow-up period. Consequently, continued booster immunization efforts may be particularly important for individuals with “waning” or “low-persistent” nAbs trajectories.

The associated factors with seroprevalence have been extensively explored. For instance, a previous study observed that participants aged 50 years had a lower seroprevalence than those aged 18–49 years (29). Consistent with this, our study found that the seroprevalence increased with age until approximately 50 years of age and declined thereafter. Additionally, both complete vaccination and prior infection with SARS-CoV-2 were positively associated with seroprevalence (30, 31). Factors associated with nAb trajectories were not explored, but the majority of them were expected. Our study revealed that younger age, female sex, full vaccination, and history of SARS-CoV-2 infection were predictors of a “high-persistent” trajectory, which indicated that these participants had a higher level and more persistent protective immunity. In line with this, previous studies also found that older participants were more likely to be “non-responders” (14). Multivariate analysis of risk factors with “waning” and “low-persistent” trajectories would contribute to identifying populations with low levels of protective immunity in the future. Future endeavors could focus on the development of risk stratification models for nAb trajectories, which may contribute to identifying high-risk populations with diminished immunity and optimizing booster immunization strategies accordingly.

Our findings highlight important public health implications, particularly for populations with waning or low-persistent neutralizing antibodies (nAbs). To maintain protective immunity, targeted monitoring and earlier booster doses should be prioritized for high-risk groups, such as older adults, men, and those with fewer vaccine doses. A tailored, data-driven approach to booster timing based on individual immune status rather than a uniform schedule could improve prevention efforts. Moreover, future studies should track long-term antibody titer changes under normal conditions after large-scale outbreaks to refine immunization strategies, especially in identifying at-risk populations and optimizing booster timing. These insights are relevant not only for China but also for global immunization policy, as countries adapt to the evolving dynamics of SARS-CoV-2 and new variants.

Our study has several strengths. First, we enrolled a large sample size to collect seroepidemiologic data and three rounds of follow-up, with a relatively high compliance rate (84.55%). Second, the large sample size allowed us to fit the antibody trajectories accurately and enabled the exploration of their associated factors. However, several limitations need to be acknowledged. Primarily, we measured nAbs against the wild strain of SARS-CoV-2 despite the Omicron variants being predominant during the study period. Although our correlation analysis showed a significant positive relationship between nAbs against the wild strain and those targeting the XBB strain of Omicron, this indirect measurement may not fully capture the immune response to Omicron and its sub-lineages, which have demonstrated immune escape properties. This limitation may affect the generalizability of our results, particularly regarding the level of protection conferred by nAbs in the real-world setting of Omicron transmission. Second, the potential factors associated with antibody trajectories were relatively limited in our study. Variables such as smoking status, body mass index, and household size, which have been shown to be associated with seroprevalence, were not extensively investigated (32, 33). Third, while nAbs serve as a robust surrogate for immunity levels, our study did not assess anti-S1 or T- and B-lymphocyte responses. The absence of data on these immune responses limits our ability to comprehensively evaluate long-term protection and memory immunity conferred by vaccination or previous infection. Fourth, the study was conducted in Zhejiang Province, which has a relatively high economic status and well-established healthcare infrastructure, leading to higher access to vaccination and better compliance. Therefore, caution should be exercised when extrapolating these findings to regions with lower socioeconomic status.

## Conclusion

In conclusion, this study provides valuable insights into the seroprevalence, nAb trajectories, and associated factors. We observed a consistently high seroprevalence of SARS-CoV-2, with three-quarters of participants exhibiting a high and persistent antibody trajectory in this highly vaccinated population. Furthermore, age, sex, COVID-19 vaccination status, and previous SARS-CoV-2 infection were all significantly associated with different antibody trajectories. In this context, monitoring protective antibody levels and promoting booster immunization strategies is crucial, especially for populations at risk of experiencing waning or low-persistent antibody trajectories.

## Data Availability

The raw data supporting the conclusions of this article will be made available by the authors, without undue reservation.
