# Supplementary material for: SARS-CoV-2 seroprevalence and antibody trajectories after easing of COVID-19 restrictions: a longitudinal study in China
Source: Front Public Health. 2024 Dec 3;12:1420993. doi: 10.3389/fpubh.2024.1420993 (PMC11650369; doi:10.3389/fpubh.2024.1420993)
Supplement: Supplementary file 1 [file Data_Sheet_1.docx]

**Additional file**

SARS-CoV-2 seroprevalence and antibody trajectories after easing COVID-19 restrictions: A longitudinal study in China

**Supplementary Methods**

*Sample size*

We calculated the sample size using the following formula:

$$n=\left( \frac{z_{\alpha/2}^{2}\times p\times\left( 1-p \right)}{\delta^{2}} \right)\times deff$$

where the *p* was the estimated seroprevalence of SARS-CoV-2 among the population and 50% was chosen in the study; Z_α/2_ is the standard normal distribution (1-α /2) × 100% quantile (in this study, the type I error probability α = 0.05, then *Z_α/2_* = 1.96). *δ* (absolute maximum allowable error) = *p-π*, is the half width of the 95% Confidence interval (CI) of the estimate, and 10% was chosen; *deff* is the ratio of the estimated variance of the actual sampling design to the estimated variance of the simple random sampling design with the same sample size, which measures the estimated efficiency of the actual sampling design. This study uses multi-stage stratified sampling, and the *deff* was 4. The calculated sample size was 385. Accounting for a 20% of loss to follow-up, the sample size was set 500 per county at the baseline.


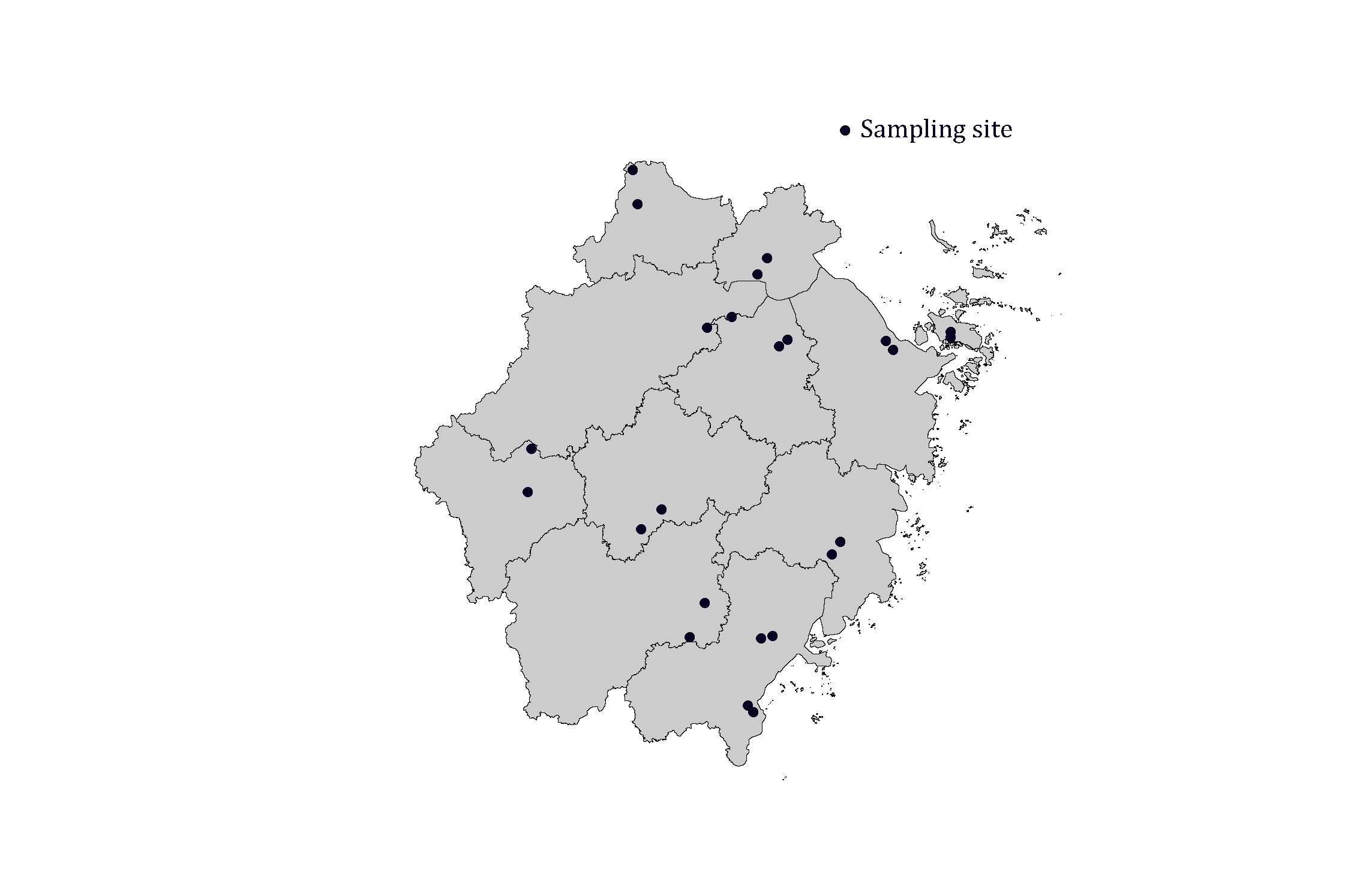


Figure S1. Sampling sites in the study in Zhejiang Province in China.

| Table S1. Study sites and enrolled sample sizes at the baseline. | | | |
| --- | --- | --- | --- |
| City | County (District) | Village (Community) | Sample size |
| Hangzhou | Xiaoshan | Bailutang | 250 |
|  |  | Dayi | 250 |
| Ningbo | Zhenhai | Qingshuipu | 256 |
|  |  | Minlian | 252 |
| Wenzhou | Lucheng | Shangsha | 259 |
|  |  | Maanchi | 250 |
|  | Longgang | Longping | 259 |
|  |  | Linjiayuan | 250 |
| Huzhou | Changxing | Huangchaodou | 251 |
|  |  | Fangxian | 254 |
| Jinhua | Wuyi | Jinhu | 280 |
|  |  | Xiashaofei | 278 |
| Zhoushan | Dinghai | Chengbei | 250 |
|  |  | Limin | 255 |
| Lishui | Qingtian | Kangfan | 263 |
|  |  | Zhoucun | 267 |
| Shaoxing | Shangyu | Minfeng | 252 |
|  |  | Changtang | 251 |
| Jiaxing | Haining | Biyun | 250 |
|  |  | Minli | 250 |
| Taizhou | Huangyan | Tayuan | 257 |
|  |  | Niansidu | 251 |
| Quzhou | Qujiang | Shangfang | 259 |
|  |  | Dongsheng | 253 |

**
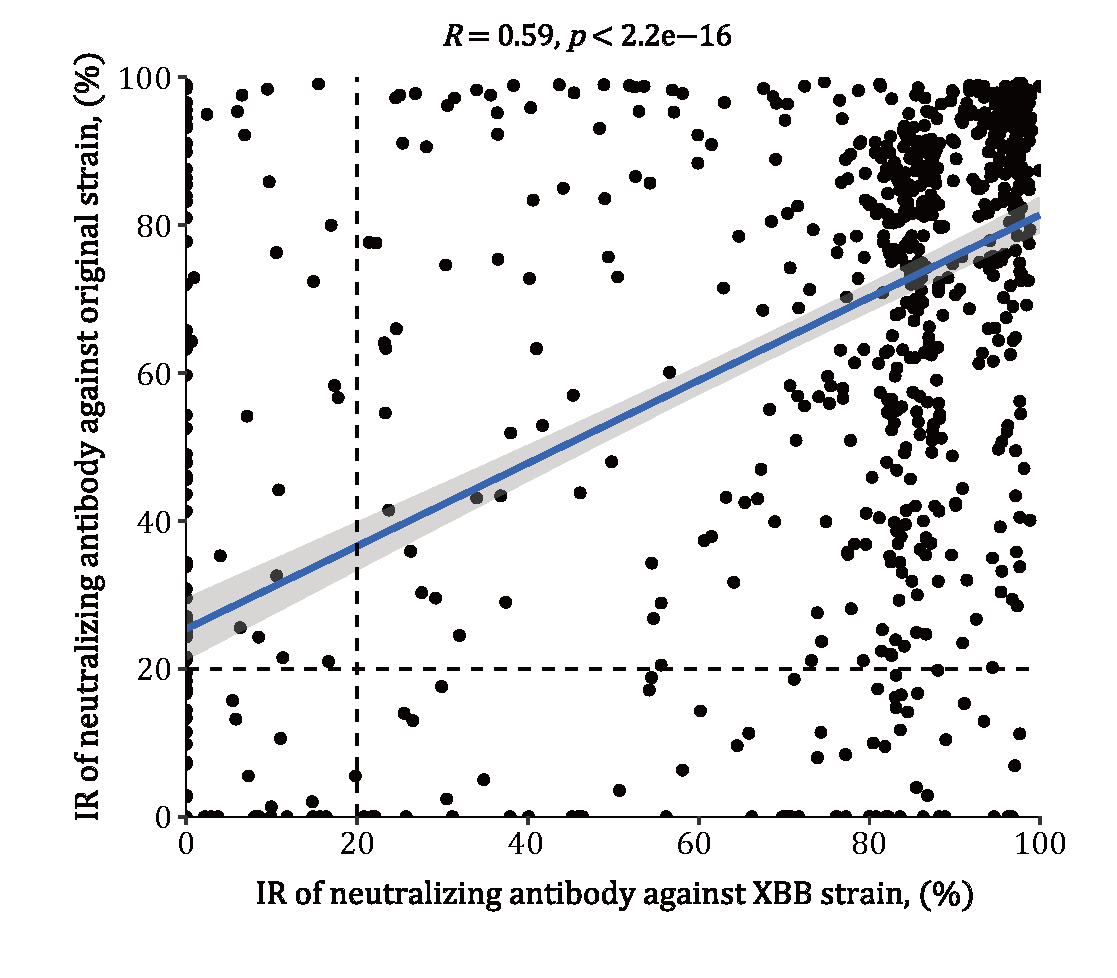
**

Figure S2. Spearman’s rank correlation of inhibition rate between SARS-CoV-2 original strain and Omicron lineages XBB strain. IR, inhibition rate

**
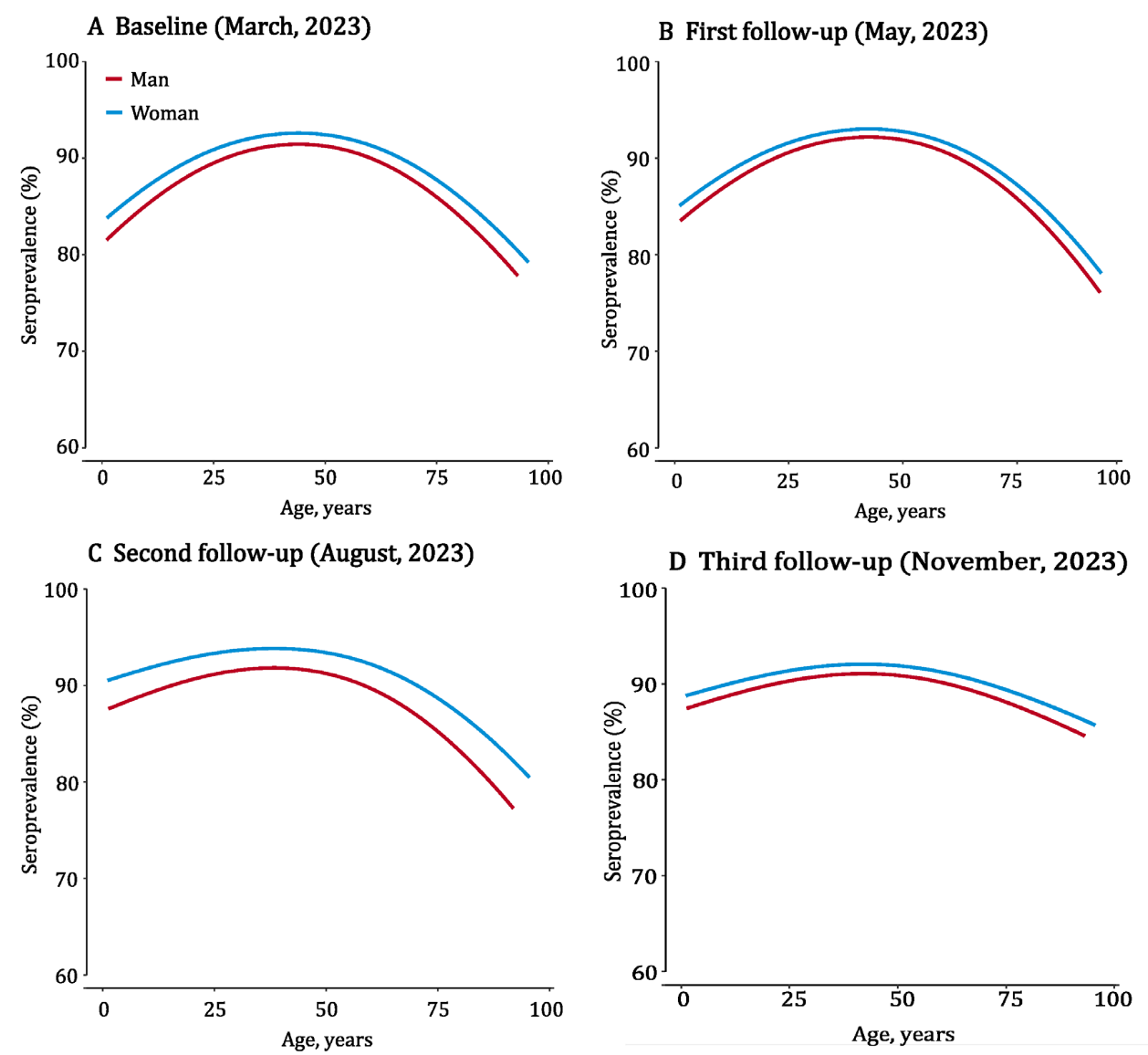
**

Figure S3. Sex-specific relationship between age and seroprevalence of SARS-CoV-2 based on generalized additive model, (A) Baseline, (B) First follow-up, (C) Second follow-up, and (D) Third follow-up.

| Table S2. Latent class growth mixture model results of fitting process. | | | | | | | |
| --- | --- | --- | --- | --- | --- | --- | --- |
| Number of latent class | Polynomial degree | Log-Lik | BIC | % Participants  per class | Mean posterior  probabilities % | Posterior  probabilities > 70% | |
| 1 | Linear | -94902 | 189855 | 100 | * | * | |
|  | Quadratic | -94671 | 189428 | 100 | * | * | |
|  | Cubic | -93762 | 187652 | 100 | * | * | |
|  |  |  |  |  |  |  | |
| 2 | Linear | -93088 | 186254 | 15.64/ 84.36 | 95.68/ 99.19 | 94.34/ 99.06 | |
|  | Quadratic | -926841 | 185483 | 15.76/ 84.24 | 99.24/ 96.69 | 98.99/ 96.09 | |
|  | Cubic | -74663 | 149540 | 62.98/ 37.02 | 98.36/ 97.07 | 98.26/ 96.78 | |
|  |  |  |  |  |  |  | |
| 3 | Linear | -93088 | 186280 | 16.01/ 83.99/ 0 | 94.54/ 68.11/ NaN | 92.19/ 0 / NaN | |
|  | Quadratic | -91567 | 183288 | 7.39/ 78.45/ 14.16 | 95.96/ 99.08/ 96.73 | 95.05/ 98.75/ 96.33 | |
|  | Cubic | -75831 | 151919 | 0 / 73.47/ 26.53 | NaN / 98.63/ 95.76 | NaN/ 98.25/ 94.92 | |
|  |  |  |  |  |  |  | |
| 4 | Linear | -93088 | 186305 | 16.32/ 83.68/ 0 /0 | 93.46/ 47.42/ NaN /NaN | 90.45/ 0 / NaN/ NaN | |
|  | **Quadratic** | **-89734** | **179657** | **8.97/ 8.08/ 75.22/ 7.74** | **93.93/ 97.50/ 99.19/ 97.58** | **92.49/ 96.43/ 98.80/ 97.26** | |
|  | Cubic | # | # | # | # | # | |
|  |  |  |  |  |  |  | |
| 5 | Linear | -93088 | 186331 | 16.64/ 0/ 0/ 83.36/ 0 | 92.19/ NaN/ NaN/ 34.20/ NaN | 88.67/ NaN/ NaN/ 0 /NaN | |
|  | Quadratic | -91567 | 183356 | 7.75/ 0/ 77.43/ 0/ 14.82 | 93.32/ NaN/ 37.78/ NaN/ 94.19 | 90.57/ NaN/ 0/ NaN 92.08 | |
|  | Cubic | # | # | # | # | # |  |
| Log-Lik, the maximum Log-Likelihood; BIC, the Bayesian information Criterion; NaN, not a number.  The best-fitting model is highlighted in bold characters.  * Not applicable.  # Models failed to converge. | | | | | | |  |

| Table S3. Characteristic of study population based on trajectories of antibody level | | | | | |
| --- | --- | --- | --- | --- | --- |
|  | High-persistent  (n=3909) | Increasing  (n=402) | Waning  (n=466) | Low-persistent  (n=420) | *P* value |
| Age, years |  |  |  |  |  |
| Median, [IQR] | 53.16 [26.80] | 56.43 [25.20] | 55.69 [22.79] | 56.22 [25.73] | <0.001 |
| <3 | 2 (0.05) | 7 (1.74) | 0 (0.00) | 13 (3.10) | <0.001 |
| 3-17 | 384 (9.82) | 40 (9.95) | 27 (5.79) | 36 (8.57) |  |
| 18-59 | 2274 (58.17) | 188 (46.77) | 259 (55.58) | 202 (48.10) |  |
| ≥60 | 1249 (31.95) | 167 (41.54) | 180 (38.63) | 169 (40.24) |  |
| Sex |  |  |  |  |  |
| Woman | 2177 (55.69) | 216 (53.73) | 237 (50.86) | 202 (48.10) | 0.008 |
| Man | 1732 (44.31) | 186 (46.27) | 229 (49.14) | 218 (51.90) |  |
| Ethnicity |  |  |  |  |  |
| Han | 3902 (99.82) | 402 (100.00) | 465 (99.79) | 419 (99.76) | 0.837 |
| Other | 7 (0.18) | 0 (0.00) | 1 (0.21) | 1 (0.24) |  |
| Living region |  |  |  |  |  |
| Urban | 1034 (26.45) | 93 (23.13) | 93 (19.96) | 98 (23.33) | 0.009 |
| Rural | 2875 (73.55) | 309 (76.87) | 373 (80.04) | 322 (76.67) |  |
| Comorbidities |  |  |  |  |  |
| 0 | 2710 (69.33) | 251 (62.44) | 305 (65.45) | 246 (58.57) | <0.001 |
| 1-2 | 1151 (29.44) | 145 (36.07) | 154 (33.05) | 166 (39.52) |  |
| ≥3 | 48 (1.23) | 6 (1.49) | 7 (1.50) | 8 (1.90) |  |
| Vaccinated against COVID-19 | |  |  |  |  |
| Unvaccinated | 39 (1.00) | 37 (9.20) | 2 (0.43) | 148 (35.24) | <0.001 |
| One dose | 15 (0.38) | 27 (6.72) | 4 (0.86) | 40 (9.52) |  |
| Two doses | 630 (16.12) | 68 (16.92) | 74 (15.88) | 52 (12.38) |  |
| ≥3 doses | 3225 (82.50) | 270 (67.16) | 386 (82.83) | 180 (42.86) |  |
| Infection with SARS-CoV-2 | |  |  |  |  |
| No | 769 (19.67) | 217 (53.98) | 104 (22.32) | 128 (30.48) | <0.001 |
| Yes | 3140 (80.33) | 185 (46.02) | 362 (77.68) | 292 (69.52) |  |
| IQR: interquartile range | | | | | |


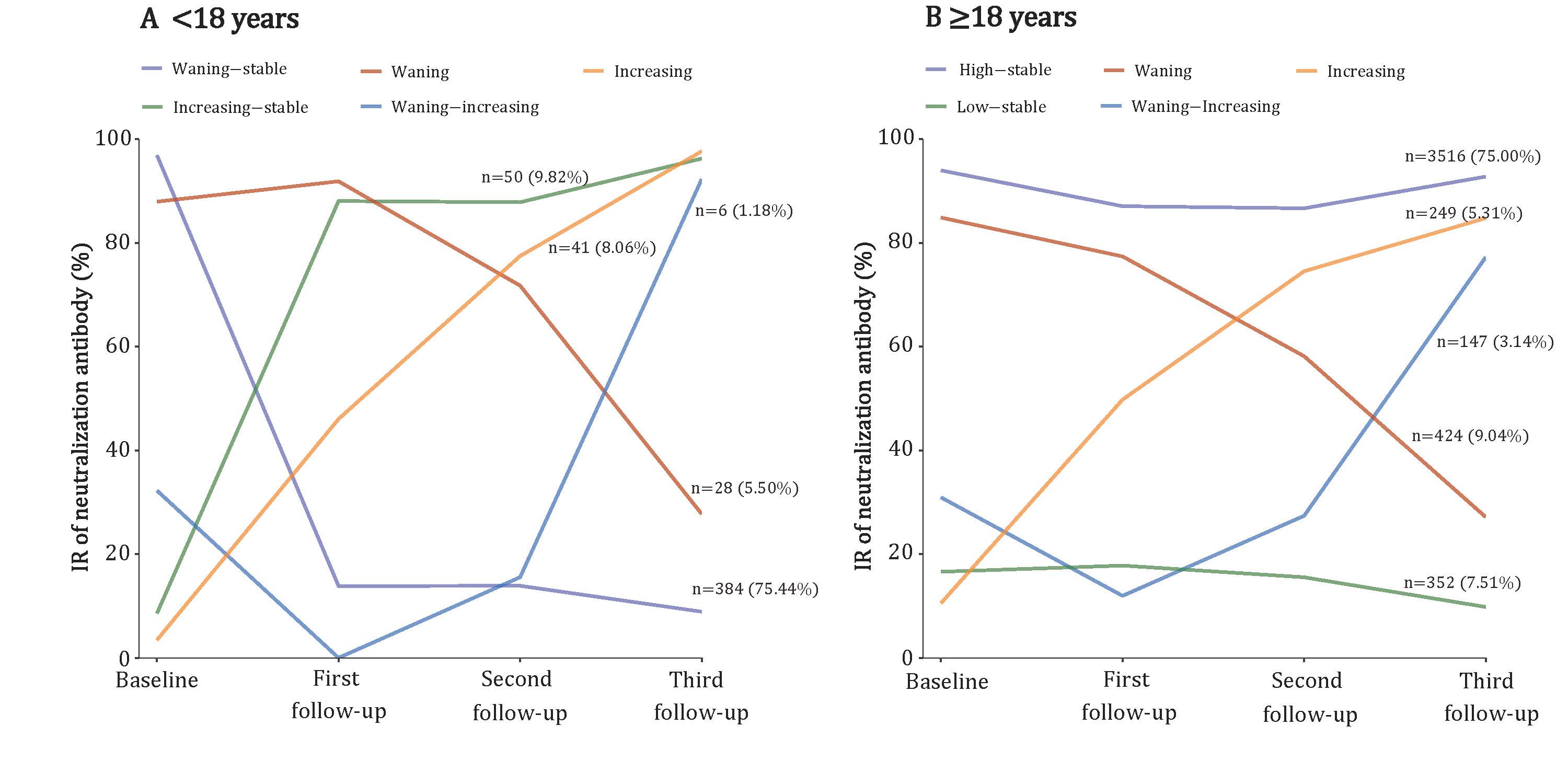


Figure S4. Stratification analysis of SARS-CoV-2 neutralizing antibody trajectories based on latent class growth mixture model.
